# Diagnostic utility of whole-genome sequencing for nephronophthisis

**DOI:** 10.1038/s41525-020-00147-8

**Published:** 2020-09-21

**Authors:** Romain Larrue, Paul Chamley, Thomas Bardyn, Arnaud Lionet, Viviane Gnemmi, Christelle Cauffiez, François Glowacki, Nicolas Pottier, Franck Broly

**Affiliations:** 1 Service de Toxicologie et Génopathies, CHU Lille, F-59000 Lille, France; 2 Univ. Lille, CNRS, Inserm, CHU Lille, UMR9020- UMR-S 1277, F-59000 Lille, France; 3 Service de Néphrologie, CHU Lille, F-59000 Lille, France; 4 Service d’Anatomo-pathologie, CHU Lille, F-59000 Lille, France

**Keywords:** Molecular medicine, Paediatric kidney disease

## Abstract

Next-generation sequencing has revolutionized the molecular diagnosis of individuals affected by genetic kidney diseases. Indeed, rapid genetic testing in individuals with suspected inherited nephropathy has not only important implications for diagnosis and prognosis but also for genetic counseling. Nephronophthisis (NPHP) and related syndromes, a leading cause of end-stage renal failure, are autosomal recessive disorders characterized by the variable presentation and considerable locus heterogeneity with more than 90 genes described as single-gene causes. In this case report, we demonstrate the utility of whole-genome sequencing (WGS) for the molecular diagnosis of NPHP by identifying two putative disease-causing intronic mutations in the *NPHP3* gene, including one deep intronic variant. We further show that both intronic variants, by affecting splicing, result in a truncated nephrocystin-3 protein. This study provides a framework for applying WGS as a first-line diagnostic tool for highly heterogeneous disease such as NPHP and further suggests that deep intronic variations are an important underestimated cause of monogenic disorders.

## Introduction

Ciliopathies represent an expanding group of heterogeneous inherited diseases primarily caused by defects in the cilium–centrosome complex^1,2^. These organelles are central to the perception of the physical environment by sensing various extracellular signals, such as growth factors, chemicals, light, and fluid flow, and are thus critical for the normal function of multiple organ systems^1,2^. Consequently, ciliary dysfunction results in a myriad of phenotypes, ranging from cystic kidney diseases and blindness to neurologic phenotypes, obesity, and diabetes^1,2^.

Nephronophthisis (NPHP [MIM: PS256100]), a rare autosomal recessive cystic kidney disease caused by mutations in genes coding for cilia-associated nephrocystin proteins, is the leading genetic cause of end-stage renal failure in the first three decades of life^1–3^. Distinct age-based clinical subtypes are recognized: infantile, juvenile, and adolescent/adult forms, according to the age of onset of renal failure^3^. To date, >90 genes, including NPHP1 (MIM: 607100) to *NPHP4* (MIM:607215), have been identified that cause NPHP and related syndromes when mutated^2,4^. At the kidney level, NPHP is characterized by an impaired renal concentrating ability, chronic tubulointerstitial nephritis, renal corticomedullary cyst formation, and progression to ESRD usually occurs before the age of 30^2,3^. As a result, renal manifestations of NPHP typically include polyuria, polydipsia with regular fluid intake at night, secondary enuresis, without evidence of proteinuria, hematuria, or cellular elements until late stage^2,3^. While in the majority of patients, NPHP occurs as an isolated kidney disease, ~10–20% NPHP-affected individuals have additional extrarenal manifestations, such as retinal degeneration, liver fibrosis as well as skeletal, neurological, and cardiac defects^1–3^.

As clinical presentation of NPHP is frequently unspecific and overlap with many other kidney diseases, accurate and timely diagnosis is essential for the clinical management of patients and genetic counseling^1,2^. Nevertheless, establishing the genetic cause of NPHP is only possible in <50% of patients^5,6^. The rapid development and application of high-throughput sequencing technologies now enable the screening of the entire genome and the identification of nearly all forms of genetic variations^7^. In this work, we report a family affected by NPHP and demonstrate the diagnostic utility of whole-genome sequencing (WGS) in particular to identify deep intronic disease-causing mutations.

## Results

### Clinical description

**Fig. 1:**
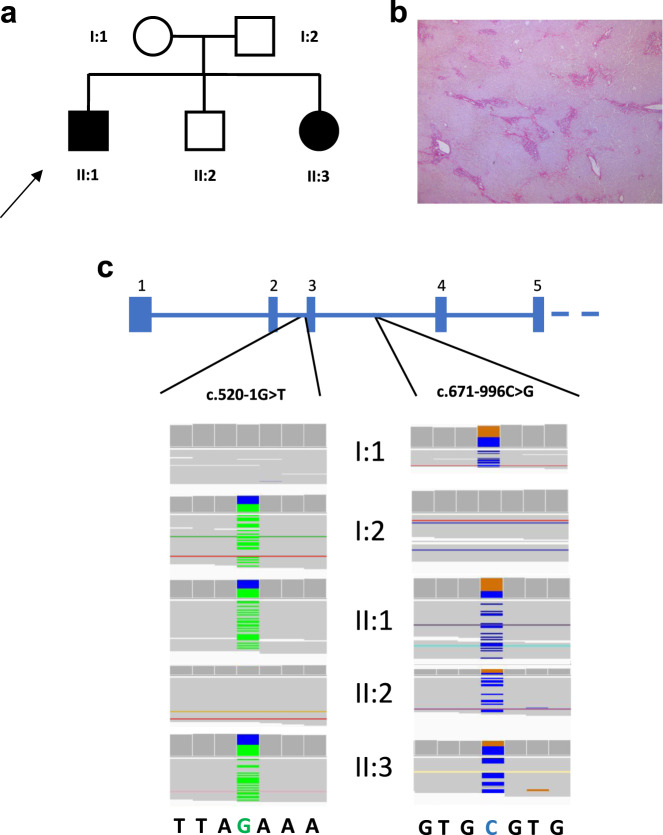
Family description. **a** Pedigree. Both individuals II:1 and II:3 are affected by NPHP. Arrow indicates the proband. **b** Representative liver section of the proband showing periportal fibrosis (Sirius Red; x20 magnification). **c** All family members were subjected to genetic analysis. Zoomed in Integrative Genomics Viewer (IGV) screenshots showing the distribution of the two pathogenic mutations identified in *NPHP3* by whole-genome sequencing (WGS) for each family member.

In this report, we describe the case of a 30-year-old patient who presented nephronophthisis with liver injury in a brotherhood of three children (Fig. 1).

At the age of nine, the proband, previously asymptomatic, presented chronic asthenia. Initial blood tests revealed anemia and terminal chronic kidney disease (CKD). Proteinuria showed a tubulointerstitial profile. Renal ultrasound imaging indicated increased echogenicity with loss of corticomedullary differentiation and identified a single cyst at the upper pole of the left kidney. No renal biopsy was performed due to the advanced stage of the disease. The fundus examination showed drusen, which remained stable during ophthalmological follow-up. Initial liver function tests showed biological cholestasis, but transaminase and bilirubin levels remained within a normal range. Genetic analysis of *NPHP1*, the only gene for which molecular testing was available at that time, was normal, and the nephropathy was thus classified as NPHP of unknown genetic origin. Renal replacement therapy was initiated with the peritoneal disease shortly after diagnosis. First kidney transplantation was performed at the age of 12, but the patient returned to hemodialysis at the age of 20, while cholestasis remained stable. At age 24, as serum transaminase levels started to rise, liver biopsies were performed and showed extensive fibrosis with ductular neogenesis (Fig. 1b). Liver function progressively worsened, and patient finally received a combined kidney–liver transplantation. Follow-up showed favorable results with normal liver function and a mean creatinine level of 1.4 mg/dL.

His younger sister was medically followed since the age of 4. Blood samples were normal, and kidney ultrasound showed hyperechogenic kidneys. At age 5, fundus examination also showed macular drusen. At age 10, her kidney function decreased rapidly leading to terminal CKD the following year. She benefited from preemptive kidney transplantation at age 12. She is now 23 years old, and follow-up shows a normal liver function.

Their younger brother, who is now 28 years of age, has also been evaluated, but shows no sign of kidney, liver, or macular disease.

### Molecular analysis

**Fig. 2:**
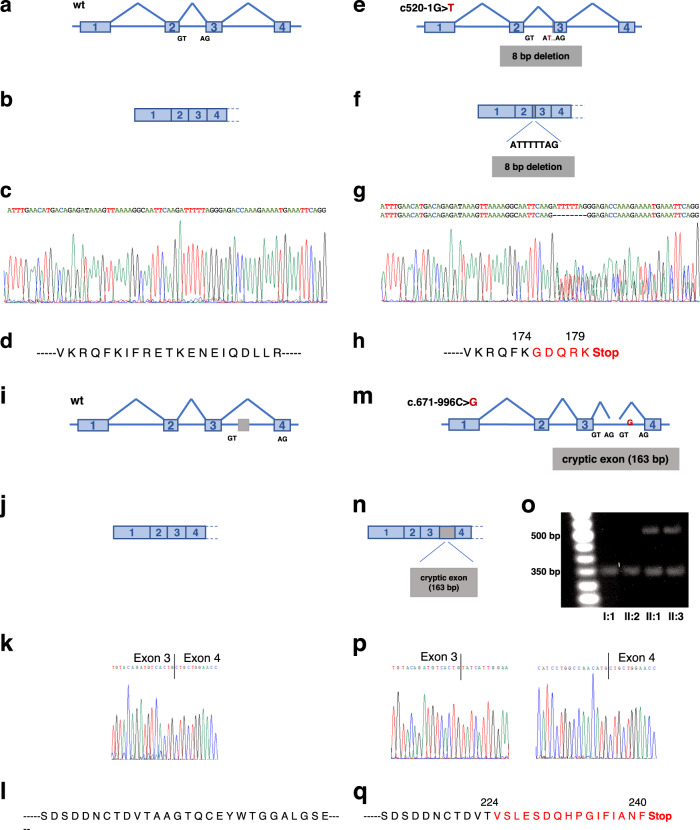
Effects of the disease-causing genetic variants on NPHP3 function. Both variants affect *NPHP3* mRNA splicing and impact protein translation through distinct mechanisms. **a**–**q** Schematic representation of the effect of the splice acceptor site mutation c.520-1G>T and the c.671-996C>G deep intronic variant on *NPHP3* mRNA splicing and its translation into protein. Wild-type sequences are indicated in panels (**a**–**d**) and (**i**–**l**), whereas sequences either bearing the c.520-1G>T mutation or the c.671-996C>G variant are shown in panels (**e**–**h**) and (**m**–**q**), respectively. **a**–**d**, **i**–**l** detail the normal splicing of *NPHP3* exon 1 to 4. **e**–**h** show that the c.520-1G>T mutation activates a cryptic splice site within exon 3 of *NPHP3*, located 8 bp downstream from the authentic 3′-splice acceptor site and leading to a frameshift and premature stop codon, which terminates protein translation 6 codons downstream (p.Ile174Glyfs*6). Representative electropherograms of both normal and mutated cDNA sequences are shown in (**c**) and (**k**), respectively. **m**–**q** show that the c.671-996C>G mutation causes aberrant *NPHP3* splicing with the insertion of a 163-nucleotide cryptic exon between exon 3 and 4 generating a frameshift and a premature stop codon at codon 240. **o** Agarose gel electrophoresis image showing aberrant *NPHP3* splicing in affected family members harboring the c.671-996C>G variant. Samples were processed in parallel. Representative electropherograms of both normal and mutated cDNA sequences are shown in (**k**) and (**p**), respectively.

A peripheral blood sample was collected from the proband (II:1), and genomic DNA was extracted. Genetic testing for NPHP was initially carried out using a dedicated targeted Next-generation sequencing (NGS) panel designed to capture the coding regions and the exon–intron boundaries of 25 genes associated with NPHP. A total of 272 variants were reviewed, and only one heterozygous mutation affecting the *NPHP3* gene (MIM:608002; RefSeq NM_153240.4) was categorized as “pathogenic” according to ACMG guidelines^8^ (Fig. 1). The remaining mutations were classified as variants of unknown significance, likely benign, or benign. This putative disease-causing variant was located in the canonical splice region of the second intron of the *NPHP3* gene at the position −1 of the acceptor splice site (NM_153240.4:c.520-1G>T; NG_008130.2:g.8288G>T; Genomic location: 3:132719145 (GRCh38); ClinVar accession number VCV000812666). Allele frequency of this mutation was below the 0.0001 thresholds for recessive gene according to the Genome Aggregation Database (gnomAD), the largest and most comprehensive public source of genetic information of individuals of different ethnic backgrounds to date. The c.520-1G>T variant was further validated using PCR and Sanger sequencing of the amplicons, confirming the diagnosis in all affected family members (II:1 and II:3) and the paternal origin of the mutation (Fig. 1). We speculated that the c.520-1G>T of the *NPHP3* gene is responsible for either the use of cryptic splice sites or exon skipping. We tested this hypothesis by RT-PCR, using total RNAs from leukocytes obtained from affected and control family members. Results of this analysis indicated that the c.520-1G>T mutation activates a cryptic splice site within exon 3, located 8 bp downstream from the authentic 3′-splice acceptor site and leading to a frameshift and premature stop codon, which terminates protein translation 6 codons downstream (p.Ile174Glyfs*6) (Fig. 2a–h).

As NPHP is inherited in an autosomal recessive manner, we reasoned that our targeted analytical strategy might have missed a deep intronic mutation or a structural variation at the *NPHP3* locus of the maternal allele. Genetic studies have recently suggested that deep intronic disease-causing mutations may be in particular more common than previously thought^9^. Therefore, we performed WGS for all family members and uncovered the deep intronic mutation NM_153240.4:c.671-996C>G (NG_008130.2:g.9528C>G; Genomic location: 3:132717905 (GRCh38); ClinVar accession number VCV000812665) located in the third intron of *NPHP3*, which was inherited from the mother and segregated with the disease in the expected autosomal recessive mode (Supplementary Table 1). These results were confirmed by Sanger sequencing. No variant at the c.671-996C>G position in *NPHP3* was identified in public databases (1000 Genomes, gnomAD, Bravo), providing further support for the hypothesis that it is likely disease-causing. To gain insight into the functional consequences induced by this deep intronic variant, we performed cDNA sequencing. This analysis revealed that the c.671-996C>G mutation was associated with the insertion of a 163-nucleotide cryptic exon within intron 3 (Genomic location: 3:132717955-132718117 (GRCh38)), located 48 bp downstream of the splice donor site and generating a frameshift as well as a premature stop codon at codon 240 (Fig. 2i–q).

## Discussion

Although individually uncommon, rare mendelian diseases collectively affect nearly 300 million people worldwide and thus represent a major public health challenge^10^. NGS technologies have revolutionized molecular diagnosis practices, and genetic testing is now on the verge of shifting from a targeted approach to sequencing an entire exome or genome^11^. In particular, WGS, the most comprehensive sequencing approach, has the great advantage of allowing the detection of all forms of genetic abnormalities in a single-laboratory workflow^7,11^. In this case report, we highlight the benefit of using WSG as a first-line diagnostic tool, especially in genetically heterogeneous diseases such as NPHP.

NPHP is an inherited disease representing one of the most common monogenic causes of end-stage renal failure in children and young adults. Suspicion of NPHP is mostly based on clinical findings, renal ultrasound imaging, and family history. Although renal ultrasonography represents a very important tool to distinguish NPHP from other inherited kidney disorders including congenital anomalies of the kidney and urinary tract (CAKUT) or nephrocalcinosis, molecular genetic analysis is currently the only method available to unequivocally identify NPHP and thus establish with certainty a diagnosis for patients and families. In this case, the proband was found to be compound heterozygous for two deleterious variants in *NPHP3*, a genotype consistent with the associated impaired liver function. Indeed, severe liver abnormalities have been described in patients harboring *NPHP3* loss-of-function mutations^12,13^. Nevertheless, molecular diagnostic testing of genetically heterogeneous diseases such as NPHP remains challenging even when applying standard NGS methods^14,15^. For instance, Braun et al., using whole-exome sequencing, failed to identify a causative single-gene mutation in ~30% of affected individuals^6^. Indeed, targeted sequencing of the protein-coding regions and the adjacent splice sites has several technical limitations, in particular for the detection of complex deletion-insertion variants, copy-number variants, or mutations located in intronic regions^7,14,15^. Our case emphasizes the importance of extending genetic screening of NPHP to sequence variations within deep intronic regions. Indeed, such pathogenic mutations usually result in the activation of non-canonical splice sites or changes in splicing regulatory elements leading to cryptic exon inclusion^16^. In our case, our initial targeted sequencing approach only identified one likely deleterious variation affecting the paternal allele, which by disrupting a canonical splice site results in an abnormal NPHP3 transcript containing a premature termination codon. The deleterious mutation inherited from the mother allele was identified in a second step by WGS. This analysis identified indeed a deep intronic variant, c.671-996C>G, that would not have been identified by standard NGS panel nor WES without specific prior knowledge. Interestingly, this intronic sequence variation most likely also causes aberrant NPHP3 splicing with the inclusion of a cryptic exon either by disrupting a splicing silencer element or creating a splicing enhancer element.

In conclusion, our study demonstrates that using WGS as a first-line test in genetically heterogeneous diseases such an NPHP is a powerful approach to increase diagnostic yields, reduce the time to diagnosis and positively impact the clinical care pathway.

## Methods

This study has been certified to be in accordance with French laws by the Institutional Review Board of Centre Hospitalier Regional Universitaire de Lille. Written informed consent was obtained from all patients.

### Whole-genome sequencing

WGS was performed with an average depth of 45× (97.2% of the whole genome was covered by at least 20× coverage). High-quality genomic DNA samples were randomly fragmented by Covaris Technology, and the fragment of 350 bp was obtained after fragment selection. The end repair of DNA fragments was performed, and an “A” base was added at the 3’-end of each strand. Adapters were then ligated to both ends of the end-repaired/dA-tailed DNA fragments, followed by amplification by ligation-mediated PCR (LM-PCR), single-strand separation, and cyclization. The rolling circle amplification (RCA) was performed to produce DNA Nanoballs (DNBs). The qualified DNBs were loaded into the patterned nanoarrays, and pair-end reads were read through on the BGISEQ-500 platform. Sequencing-derived raw image files were processed by BGISEQ-500 base calling Software (v1) with default parameters, and the sequence data of each individual was generated as paired-end reads.

### Bioinformatics analysis

Data of each sample were mapped to the human reference genome (GRCh37/HG19) using Burrows–Wheeler Aligner software (v0.7.17). Variant calling was performed using Genome Analysis Toolkit. Variant quality score recalibration method, which uses machine learning to identify annotation profiles of variants that are likely to be real, was applied to get high-confident variant calls. In order to decrease the noise of sequencing data, data filtering was performed and included (1) removing reads containing sequencing adapter; (2) removing reads whose low-quality base ratio (base quality less than or equal to 5) is >50%; (3) removing reads whose unknown base ratio is >10%. Statistical analysis of the data and downstream bioinformatics analysis were performed on this filtered, high-quality data. The SnpEff tool was applied to perform: (1) gene-based annotation: identify whether variants cause protein-coding changes and the amino acids that are affected (2) filter-based annotation: identify variants that are reported in dbSNP v141, or identify the subset of variants with MAF < 1% in the 1000 Genome Project, or identify the subset of coding non-synonymous SNPs with SIFT score < 0.05, or find intergenic variants with GERP++score > 2, or many other annotations on specific mutations.

### Reporting summary

Further information on research design is available in the Nature Research Reporting Summary linked to this article.

## Supplementary information

Supplemental material

Reporting Summary

## Data Availability

WGS data are deposited in SRA (Sequence Read Archive) public repository (Accession number: PRJNA649865). All other relevant data are available from the authors upon request.
