# Supplementary material for: Diagnostic utility of whole-genome sequencing for nephronophthisis
Source: NPJ Genom Med. 2020 Sep 21;5:38. doi: 10.1038/s41525-020-00147-8 (PMC7506526; doi:10.1038/s41525-020-00147-8)

**Supplemental Table 1** : List of the rare variants (Allelic frequency below 0.01) identified by whole genome sequencing for the 25 genes associated with nephronophthisis.

| Gene | Transcript HGNC | Chr | Coordinate | Read Depth | Consequence | HGVSc | HGVSp | rs number | Allelic Frequency (GnomAD) |
|---|---|---|---|---|---|---|---|---|---|
| AHI1 | AHI1 | 6 | 135677850 | 49 | intron variant | NM_017651.4:c.3165+1420G>T | | rs991356050 | 0,000064 |
| AHI1 | AHI1 | 6 | 135662322 | 43 | intron variant | NM_017651.4:c.3165+16948T>G | | rs949993157 | 0,000064 |
| AHI1 | AHI1 | 6 | 135700226 | 56 | intron variant | NM_017651.4:c.3109+15688A>G | | rs118113442 | 0,008950 |
| AHI1 | AHI1 | 6 | 135776462 | 57 | intron variant | NM_017651.4:c.1344+410G>C | | rs116988199 | 0,009841 |
| ATXN10 | ATXN10 | 22 | 46223009 | 40 | intron variant | NM_013236.3:c.1238-15828_1238-15819delACACACACAC | | | |
| ATXN10 | ATXN10 | 22 | 46101725 | 42 | intron variant | NM_013236.3:c.647+2998G>A | | rs147527047 | 0,008571 |
| CC2D2A | CC2D2A | 4 | 15473415 | 42 | intron variant | NM_001080522.2:c.-52-1431G>A | | | |
| CC2D2A | CC2D2A | 4 | 15576762 | 40 | intron variant | NM_001080522.2:c.3771+813T>A | | | |
| CC2D2A | CC2D2A | 4 | 15578350 | 57 | intron variant | NM_001080522.2:c.3771+2401C>T | | rs537137500 | 0,000191 |
| CC2D2A | CC2D2A | 4 | 15570700 | 42 | intron variant | NM_001080522.2:c.3399-216G>A | | rs141875761 | 0,004617 |
| CC2D2A | CC2D2A | 4 | 15599864 | 36 | intron variant | NM_001080522.2:c.4496+776T>C | | rs571905411 | 0,006814 |
| CC2D2A | CC2D2A | 4 | 15561718 | 43 | intron variant | NM_001080522.2:c.2923-436G>A | | rs562301411 | 0,007581 |
| CEP290 | CEP290 | 12 | 88461375 | 45 | intron variant | NM_025114.3:c.6135+924A>G | | | |
| CEP290 | CEP290 | 12 | 88505426 | 51 | intron variant | NM_025114.3:c.2217+45T>C | | rs45461003 | 0,007123 |
| CEP83 | CEP83 | 12 | 94711739 | 43 | intron variant | NM_016122.2:c.1708-4946G>A | | rs186132204 | 0,000733 |
| CEP83 | CEP83 | 12 | 94852290 | 34 | intron variant | NM_016122.2:c.-155+1043C>T | | rs141695447 | 0,006733 |
| DCDC2 | DCDC2 | 6 | 24198532 | 38 | intron variant | NM_001195610.1:c.1023+6698C>T | | rs772350286 | 0,000127 |

| | | | | | | | | | |
|---|---|---|---|---|---|---|---|---|---|
| DCDC2 | DCDC2 | 6 | 24330238 | 50 | intron variant | NM_001195610.1:c.348+23559A>G | | rs565471349 | 0,001592 |
| DCDC2 | DCDC2 | 6 | 24232232 | 40 | intron variant | NM_001195610.1:c.923-26906_923-26903dupGTGT | | rs9467088 | 0,008403 |
| DCDC2 | DCDC2 | 6 | 24184088 | 45 | intron variant | NM_001195610.1:c.1024-5228G>A | | rs147329271 | 0,009205 |
| GLIS2 | GLIS2 | 16 | 4382383 | 34 | synonymous variant | NM_032575.2:c.102T>G | NM_032575.2:c.102T>G(p.=) | | |
| KRTCAP3,IFT172 | IFT172 | 2 | 27669137 | 50 | missense variant | NM_015662.1:c.4745T>C | NP_056477.1:p.Ile1582Thr | rs61742074 | 0,007594 |
| INVS | INVS | 9 | 102943603 | 39 | intron variant | NM_014425.3:c.274-44741G>A | | rs770333825 | 0,000096 |
| INVS | INVS | 9 | 103007686 | 40 | intron variant | NM_014425.3:c.907-1212C>T | | rs559486488 | 0,003475 |
| IQCB1 | IQCB1 | 3 | 121520657 | 33 | intron variant | NM_001023570.2:c.588-2438_588-2437dupAA | | rs5852277 | 0,000000 |
| IQCB1 | IQCB1 | 3 | 121539249 | 46 | intron variant | NM_001023570.2:c.393+5648dupA | | rs755393939 | 0,000382 |
| IQCB1 | IQCB1 | 3 | 121504284 | 48 | intron variant | NM_001023570.2:c.1278+2847A>G | | rs139185423 | 0,003728 |
| MAPKBP1 | MAPKBP1 | 15 | 42108933 | 36 | intron variant | NM_001128608.1:c.1603+85C>T | | rs185338746 | 0,000319 |
| NPHP3-ACAD11,NPHP3 | NPHP3 | 3 | 132436749 | 44 | intron variant | NM_153240.4:c.671-996C>G | | | |
| NPHP3-ACAD11,NPHP3 | NPHP3 | 3 | 132437989 | 41 | splice acceptor variant | NM_153240.4:c.520-1G>T | | rs759262253 | 0,000004 |
| NPHP4 | NPHP4 | 1 | 6038236 | 35 | intron variant | NM_015102.3:c.279+94G>A | | rs550045403 | 0,000385 |
| RPGRIP1L | RPGRIP1L | 16 | 53718888 | 42 | intron variant | NM_015272.2:c.776+1457A>G | | rs144762840 | 0,008431 |
| SDCCAG8 | SDCCAG8 | 1 | 243468775 | 48 | intron variant | NM_006642.3:c.740+698_740+701delGAGT | | rs141220766 | 0,003648 |
| TMEM67 | TMEM67 | 8 | 94817238 | 51 | intron variant | NM_153704.5:c.2439+132T>C | | | |
| TTC21B | TTC21B | 2 | 166735443 | 38 | intron variant | NM_024753.4:c.3805+1746G>A | | | |
| TTC21B | TTC21B | 2 | 166739568 | 51 | intron variant | NM_024753.4:c.3684+736C>T | | rs562591100 | 0,000032 |

| | | | | | | | | |
|---|---|---|---|---|---|---|---|---|
| TTC21B | TTC21B | 2 | 166790267 | 40 | intron variant | NM_024753.4:c.711-700G>A | rs113701367 | 0,005160 |
| TTC21B | TTC21B | 2 | 166747978 | 40 | intron variant | NM_024753.4:c.2951-480G>A | rs1982056 | 0,005193 |
| WDR19 | WDR19 | 4 | 39190238 | 33 | intron variant | NM_025132.3:c.165-1021delA | rs1207091579 | 0,007504 |
| XPNPEP3 | XPNPEP3 | 22 | 41316878 | 40 | intron variant | NM_022098.3:c.1056-1459C>T | | |
| XPNPEP3 | XPNPEP3 | 22 | 41318788 | 65 | intron variant | NM_022098.3:c.1236+271A>G | rs921253835 | 0,000032 |
| XPNPEP3 | XPNPEP3 | 22 | 41318589 | 56 | intron variant | NM_022098.3:c.1236+72C>G | rs921286516 | 0,000032 |
| ZNF423 | ZNF423 | 16 | 49847410 | 41 | intron variant | NM_015069.3:c.16+9171G>T | rs947264565 | 0,000032 |
| ZNF423 | ZNF423 | 16 | 49847411 | 40 | intron variant | NM_015069.3:c.16+9170T>A | rs1044705345 | 0,000032 |
| ZNF423 | ZNF423 | 16 | 49822431 | 49 | intron variant | NM_015069.3:c.76+967G>C | rs770926931 | 0,000064 |
| ZNF423 | ZNF423 | 16 | 49655856 | 42 | intron variant | NM_015069.3:c.3577+4225T>C | rs555247013 | 0,000798 |
| ZNF423 | ZNF423 | 16 | 49558167 | 61 | intron variant | NM_015069.3:c.3710-517A>G | rs118188965 | 0,002039 |
| ZNF423 | ZNF423 | 16 | 49552625 | 31 | intron variant | NM_015069.3:c.3825+4910T>C | rs191113819 | 0,002070 |
| ZNF423 | ZNF423 | 16 | 49850570 | 45 | intron variant | NM_015069.3:c.16+6011G>A | rs143100685 | 0,009110 |

**Supplemental Figure** : Uncropped gel electrophoresis image related to Figure 2o.

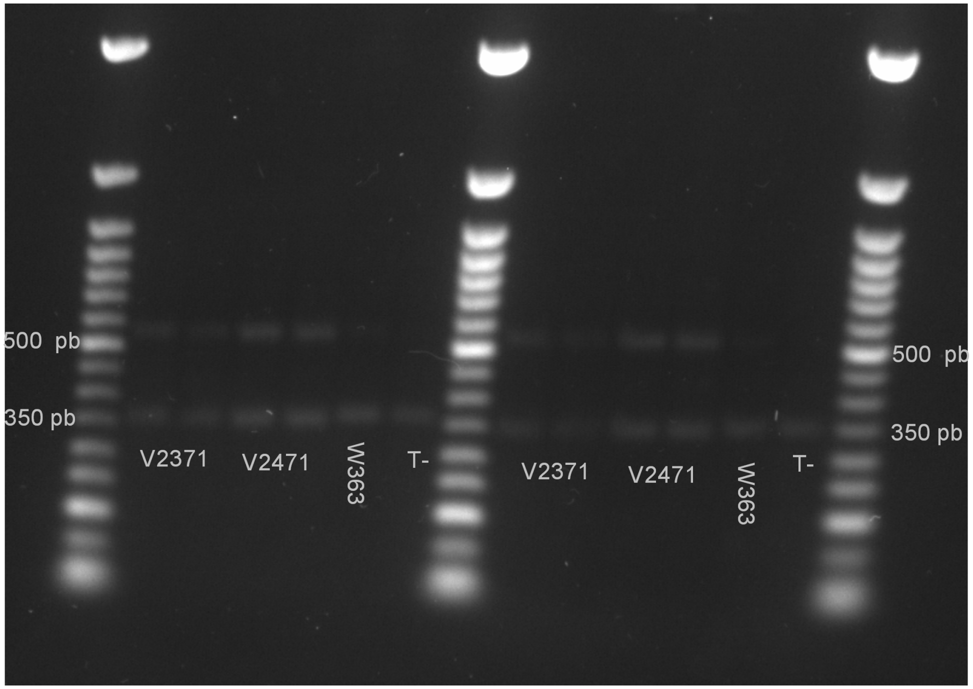

Supplement: Supplementary file 1 — Supplemental material [file 41525_2020_147_MOESM1_ESM.pdf]
